# Paclitaxel improved anti-L1CAM lutetium-177 radioimmunotherapy in an ovarian cancer xenograft model

**DOI:** 10.1186/s13550-014-0054-2

**Published:** 2014-10-03

**Authors:** Dennis Lindenblatt, Eliane Fischer, Susan Cohrs, Roger Schibli, Jürgen Grünberg

**Affiliations:** Center for Radiopharmaceutical Sciences ETH-PSI-USZ, Paul Scherrer Institute, 5232 Villigen PSI, Switzerland; Department of Chemistry and Applied Biosciences, ETH Zürich, 8093 Zürich, Switzerland

**Keywords:** ^177^Lu-radioimmunotherapy, Paclitaxel, Combination therapy, Ovarian carcinoma, L1CAM, mAb chCE7

## Abstract

**Background:**

Today’s standard treatment of advanced-stage ovarian cancer, including surgery followed by a paclitaxel-platinum-based chemotherapy, is limited in efficacy. Recently, we could show that radioimmunotherapy (RIT) with ^177^Lu-labelled anti-L1 cell adhesion molecule (L1CAM) monoclonal antibody chCE7 is effective in ovarian cancer therapy. We investigated if the efficacy of anti-L1CAM RIT can be further improved by its combination with paclitaxel (PTX).

**Methods:**

*In vitro* cell viability and cell cycle arrest of human ovarian cancer cells were assessed upon different treatment conditions. For therapy studies, nude mice (*n* = 8) were injected subcutaneously with IGROV1 human ovarian carcinoma cells and received a single dose of 6 MBq ^177^Lu-DOTA-chCE7 alone or in combination with 600 μg PTX (31.6 mg/kg). Tumour growth delay and survival were determined. To investigate whether PTX can influence the tumour uptake of the radioimmunoconjugates (RICs), a biodistribution study (*n* = 4) and SPECT/CT images were acquired 120 h post injections of 2 MBq ^177^Lu-DOTA-chCE7 alone or in combination with 600 μg PTX.

**Results:**

Lu-DOTA-chCE7 in combination with PTX revealed a significantly decreased cell viability of ovarian carcinoma cells *in vitro* and was effective in a synergistic manner (combination index < 1). PTX increased the RIT efficacy by arresting cells in the radiosensitive G2/M phase of the cell cycle 24 h post treatment start. *In vivo* combination therapy including ^177^Lu-DOTA-chCE7 and PTX resulted in a significantly prolonged overall survival (55 days vs. 18 days/PTX and 29 days/RIT), without weight loss and/or signs of toxicity. Biodistribution studies revealed no significant difference in tumour uptakes of ^177^Lu-DOTA-chCE7 72 h post injection regardless of an additional PTX administration.

**Conclusions:**

Combination of anti-L1CAM ^177^Lu-RIT with PTX is a more effective therapy resulting in a prolonged overall survival of human ovarian carcinoma-bearing nude mice compared with either monotherapy. The combination is promising for future clinical applications.

**Electronic supplementary material:**

The online version of this article (doi:10.1186/s13550-014-0054-2) contains supplementary material, which is available to authorized users.

## Background

With an estimate of 21,980 new cases in the US alone, ovarian carcinoma (OC) represents the fifth most common cause of cancer deaths in female population in 2014 [1]. Late diagnosis due to missing clinical symptoms or diagnostic markers results in poor prognosis for patients that have often developed late-stage ovarian cancer, including widespread metastases at the time of diagnosis [2,3]. Today’s front-line therapies, including surgery followed by a paclitaxel-platinum treatment, fail to cure late-stage OC. However, the 10-year survival rate is 40% to 50% with stage-related survival of 73% to 92% for stage I, 45% to 55% for stage II, 21% for stage III and less than 6% for stage IV patients [3,4]. Therefore, alternative treatment strategies are subject of intense research.

Besides conventional chemotherapy, numerous monoclonal antibodies (mAbs) have been developed for targeted therapies for the future management of ovarian cancer [5]. Although preclinical studies showed promising results, clinical administration of mAbs as monotherapies or when combined with other treatment modalities showed only limited clinical efficacy in OC patients [6-9]. The reasons for restricted mAb activities are not obvious. However, studies will benefit from larger trials and appropriate patient selections to better define the effectiveness of mAb-based therapies.

In order to improve the efficacy of mAb-based therapies, radioimmunotherapy (RIT) is considered to be an attractive strategy for the treatment of OC [10]. While RIT has only a limited efficacy treating larger solid tumours due to insufficient dose delivery, it is a suitable therapy option for small-volume disseminated tumour nodules that frequently occur after surgery of the primary tumours of the ovary [11,12]. However, only one ^90^Y-labelled radioimmunconjugate (^90^Y-muHMFG1) for the treatment of OC has advanced to a clinical phase III trial. Unfortunately, no improvement in extending survival or time to relapse could be achieved in particular due to a missing dosimetric approach. Thereby, retrospective analysis revealed that tumour absorbed doses had been too low [13,14].

The use of the high-energy-emitting radionuclide ^90^Y and the application of a non-internalising antibody (anti-MUC1) are considered further reasons for the limited clinical outcome.

The L1 cell adhesion molecule (L1CAM) was originally described as a protein of the nervous system and is highly expressed on numerous tumours such as neuroblastoma [15], colon carcinoma [16], melanoma [17], pancreatic adenocarcinoma [18] and ovarian carcinoma [19]. Its expression in cancer is correlated with increased cell proliferation, migration, angiogenesis as well as apoptosis protection [15,19,20]. Therefore, L1CAM is a promising target for novel therapies [21-24].

chCE7 is a chimeric monoclonal antibody that is directed against the L1CAM cell surface antigen. mAb chCE7 binds with high affinity (*K*_D_ ≈ 10^−10^ mol/l) near an RGD sequence in the sixth IgG-like domain of L1CAM, inhibiting tumour cell growth *in vitro* and *in vivo* [15,25,26]. The antibody-antigen complex internalises into the targeted cell through endocytosis. We demonstrated that a ^177^Lu-labelled variant of mAb chCE7 showed high efficacy in a xenograft model of disseminated ovarian carcinoma [25].

Preclinical studies have demonstrated that combined treatments including RIT and radiosensitising taxanes such as paclitaxel (PTX) can be advantageous compared to monotherapies [27-29]. PTX belongs to the group of microtubule-stabilising agents and induces apoptosis and arrest of tumour cells in the radiosensitive G2/M phase of the cell cycle based on suppression of microtubule dynamics. Furthermore, it was shown that PTX influences the tumour microenvironment, resulting in reoxygenation of the tumour potentially providing radiosensitising effects [30,31].

In this study, we investigated whether the efficacy of previously developed anti-L1CAM ^177^Lu-RIT against ovarian carcinoma can be further increased by its combination with the radiosensitising taxane PTX.

## Methods

### Cell culture and antibody formats

IGROV1 human ovarian cancer cells were kindly provided by Dr. Cristina Müller (Center for Radiopharmaceutical Sciences, Paul Scherrer Institute) and analysed by STR profiling (DSMZ, Braunschweig, Germany). IGROV1 cells were maintained in a humidified atmosphere containing 5% CO_2_ in RPMI 1640 medium at 37°C. The medium was supplemented with 10% fetal calf serum (FCS), 2 mM glutamine, 100 units/ml penicillin, 100 μg/ml streptomycin and 0.25 μg/ml fungizone (BioConcept, Allschwil, Switzerland). mAb chCE7 is a IgG1-subtype chimeric monoclonal antibody (human κ light chain and human γ1 heavy chain). It was produced in HEK293 cells and purified from cell culture supernatant using a protein G-Sepharose column (GE Healthcare, Glattbrugg, Switzerland) as described by Grünberg et al. [32]. An unspecific isotype-matched IgG was used as a control for experiments.

### Ligand substitution and antibody radiolabelling

Ligand substitution was performed as previously described by Fischer et al. [25]. For ligand conjugation, the molar excess of p-SCN-Bn-DOTA (Macrocyclics, Dallas, TX, USA) was adapted individually for each antibody to achieve similar DOTA ligands to mAb ratios. The reaction mixture was adjusted to pH 9 to 10 using a saturated Na_3_PO_4_ solution and was incubated for 16 h at 4°C. Excess ligands were removed and buffer was exchanged into 0.25 M CH_3_COONH_4_ (pH 5.5) using a NAP-5 column (GE Healthcare, Glattbrugg, Switzerland). Immunoconjugates were stored at −80°C.

The average number of coupled chelators per mAb was determined by mass spectrometry as previously described [25]. ^177^Lu (ITG, Garching, Germany) was utilised for radiolabelling 1 to 3 days post calibration date. Briefly, a reaction mixture containing 250 to 900 μg of the immunoconjugates and 200 to 600 MBq ^177^Lu was incubated in 0.25 M CH_3_COONH_4_ buffer (pH 5.5) for 1 h at 37°C.

After incubation, EDTA was added to a final concentration of 5 mM for 5 min in order to complex free lutetium. Radioimmunoconjugates (RICs) were purified via FPLC size exclusion chromatography on a Superose 12 column (GE Healthcare, Glattbrugg, Switzerland) in phosphate-buffered saline (PBS) with a flow rate of 0.5 ml/min. Both radiolabelled chCE7 and unspecific control IgG eluted at a retention time of 21 min. In order to test the stability of ^177^Lu-labelled antibodies, RICs were incubated in human plasma at 37°C and analysed by FPLC size exclusion chromatography on a TSKgel G3000Wxl column (Tosoh Bioscience, Stuttgart, Germany). The flow rate of the mobile phase (0.3 M NaCl, 0.05 M Na_2_HPO_4_, pH 6.2) was set to 1 ml/min (Additional file 1: Figure S1).

### FACS cell cycle analysis upon PTX treatment

For cell cycle analysis, IGROV1 cells were seeded in a six-well plate (0.75 × 10^5^/well) and incubated for 24 h. The medium was removed and cells were incubated with the accordant ½ half-maximal inhibitory concentration (½ IC_50_, 5 nM) or IC_50_ (10 nM) of PTX for 24 h at 37°C. PTX ½ IC_50_ was calculated based on the experimentally determined IC_50_ value using a 3-(4,5-dimethylthiazol-2-yl)-2,5-diphenyltetrazolium bromide (MTT) cell viability assay (Additional file 1: Figure S2). Afterwards, cells were washed with PBS, detached and fixed in 70% ethanol (24 h, −20°C). After additional washing with PBS, cells were incubated with 0.5 μg/ml propidium iodide (PI) solution (Sigma-Aldrich, Buchs, Switzerland) for 40 min at room temperature (RT) and analysed by flow cytometry. All results were evaluated with FlowJo software (Tree Star, Ashland, OR, USA, version 10).

### *In vitro* cell viability assay

In order to determine the ^177^Lu-DOTA-chCE7 concentrations necessary to reduce cell viability to 50% (IC_50_), IGROV1 cells were seeded in a 96-well plate and incubated for 24 h at 37°C. After adhesion, cells receiving combination treatment were incubated with the accordant ½ IC_50_ (5 nM) or IC_50_ (10 nM) PTX concentrations for 24 h at 37°C in order to maximise the amount of cells being arrested in the G2/M phase of the cell cycle. Cells were then washed with PBS and treated with 100 μl (0.02 to 42 MBq/ml) ^177^Lu-DOTA-chCE7 for 4 h on ice. Subsequently, cells were washed and incubated in culture medium at 37°C. Cell viability was determined when 20 μl of filtered MTT solution (5 mg/ml, Sigma-Aldrich) was added to each well followed by incubation for 2 h protected from light. The medium was removed and the formed formazan crystals were dissolved in 200 μl dimethyl sulfoxide (DMSO). The absorbance (OD) was determined at a wavelength of 560 nm in a microplate reader (Victor X3, PerkinElmer, Waltham, MA, USA). Results are expressed as percentage of viable cells compared to the control.

### *In vivo* therapy studies

All animal experiments were approved by the cantonal committee on animal experiments and permitted by the responsible cantonal authorities (permission numbers 75528 and 75535). The studies were conducted in compliance with the Swiss laws on animal protection. For survival studies, groups of eight female CD1 nude mice (Charles River, Sulzfeld, Germany, 5 weeks old) were injected subcutaneously (s.c.) with 7 × 10^6^ IGROV1 cells (100 μl, in sterile PBS) into the right flank. Eight days post tumour cell inoculation, therapy experiments started (mean tumour volume = 60 ± 30 mm^3^). Mice were injected with a) 6 MBq (50% maximum tolerated activity (MTA), 25 μg, 100 μl) ^177^Lu-DOTA-chCE7, b) 6 MBq ^177^Lu-DOTA-control IgG (25 μg, 100 μl) or c) PBS into the tail vein. MTA of ^177^Lu-DOTA-chCE7 was determined elsewhere [33]. Twenty-four hours later, groups that should receive the combination therapy or PTX alone were injected intraperitoneally (i.p.) with 600 μg PTX (31.6 mg/kg, clinical formulation; Taxol, Bristol-Myers Squibb, Zürich, Switzerland, 1:3 dilution with PBS; 300 μl). Since the maximum tumour uptake of ^177^Lu-DOTA-chCE7 is reached at 48 to 72 h after injection of RIC (i.v.), PTX was administered 24 h post RIT in order to synchronise the maximum tumour uptake of the radiolabeled mAb and PTX-induced cell cycle arrest in the G2/M phase. Concentration was chosen based on previous experiments determining 600 μg/mouse as a reliable dosage for PTX administration in combination with RIT [34,35].

Animals were examined two to three times a week and weighed, and tumour volumes were measured with *V* = (*A* × *B*^2^)/2, where *A* is the smaller diameter and *B* the wider diameter of the tumour. The relative tumour volume (RTV) was calculated with *V*_*x*_/*V*_0_ (*V*_*x*_ = tumour volume at given time, *V*_0_ = tumour volume at therapy starting point). Relative body weight (RBW) was measured with *W*_*x*_/*W*_0_ (*W*_*x*_ = body weight at given time, *W*_0_ = body weight at therapy starting point). Animals were euthanised if the tumour volume exceeded 1,000 mm^3^ or observed weight loss was greater than 20%. In order to avoid bias in the test results, treatment information was blinded to the tester during the course of the therapy.

### Biodistribution studies

For biodistribution studies, groups of four female CD1 nude mice (Charles River, Sulzfeld, Germany) were injected s.c. with 7 × 10^6^ IGROV1 cells at the age of 5 weeks. Fourteen days post tumour cell inoculation, 0.85 MBq (25 μg, 100 μl) ^177^Lu-DOTA-chCE7 was injected into the tail vein. Twenty-four hours later, mice received either 600 μg PTX (31.6 mg/kg, clinical formulation; Taxol, Bristol-Myers Squibb, Zürich, Switzerland, 1:3 dilution with PBS; 300 μl) i.p. or 300 μl PBS i.p. Control mice were injected with 0.85 MBq ^177^Lu-DOTA-control IgG (25 μg, 100 μl). Mice were sacrificed 72 h post RIC administration, and organs as well as tumours were weighed and counted for radioactivity in a gamma counter (COBRA II, Packard Bioscience, Meriden, CT, USA). Results are expressed as percentage of the injected activity per gram of tissue weight (%IA/g).

### SPECT/CT imaging studies

SPECT/CT imaging studies were performed in a NanoSPECT/CT system (Bioscan, Washington, DC, USA). ^177^Lu-DOTA-chCE7 (6 MBq, 25 μg, 100 μl) was injected into the tail vein of tumour-bearing nude mice 24 h prior to i.p. administration of 600 μg (31.6 mg/kg) PTX or PBS. Control mice received ^177^Lu-DOTA-control IgG. SPECT/CT scans were performed 96 h (^177^Lu-DOTA-control IgG) and 120 h (^177^Lu-DOTA-chCE7, ^177^Lu-DOTA-chCE7 + PTX) after RIC administration. SPECT data were reconstructed by HighSPECT software (ver. 1.4.3049, Scivis). Reconstruction of CT data, fusion with SPECT data and analysis were performed by InVivoScope postprocessing software (ver. 1.44, Bioscan, Washington, DC, USA).

### Statistical analysis

Statistical analysis of the survival experiment was performed via a log-rank test. Bonferroni correction was used to determine statistical significance for multiple comparisons. Significance was defined as *p* < 0.0083. Student’s *t* test (unpaired, two-tailed) was used for comparison of tumour volumes and biodistribution experiments. Statistical significance was defined as *p* < 0.05. *In vitro* data was analysed via combination index calculations (CI = (*C*_A,*x*_/Ic_*x*,A_) + (*C*_B,*x*_/Ic_*x*,B_)). Thereby, concentrations required to produce a given effect are determined for drug A (Ic_*x*,A_) and drug B (Ic_*x*,B_). *C*_A*,x*_ and *C*_B,*x*_ are the concentrations of A and B contained in combination that provide the same effect. Synergy is determined for CI < 1, additivity for CI = 1 and antagonism for CI > 1 [36].

## Results

### Ligand substitution and antibody radiolabelling

Mass spectroscopic analysis of the DOTA-to-mAb ratios for mAb chCE7 and the control IgG revealed that an average of 2.7 to 3.1 (chCE7) or 3.1 (control IgG) chelators were coupled to an intact antibody molecule. Specific activity obtained upon ^177^Lu labelling was 240 to 600 MBq/mg protein. Immunoreactivity was proven by the Lindmo method (50% to 87%).

### PTX treatment induces G2/M phase arrest

**Figure 1:**
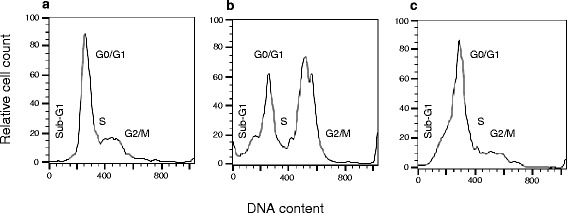
**FACS analysis of PTX pre-treated IGROV1 cells. (a)** Untreated control, **(b)** incubated with 10 nM PTX (IC_50_) or **(c)** incubated with 5 nM PTX (½ IC_50_) for 24 h at 37°C.

Figure 1 shows fluorescence-activated cell sorting (FACS) analysis histograms after IGROV1 cells have been treated with ½ IC_50_ (5 nM) or IC_50_ (10 nM) PTX concentrations for 24 h. An increased amount of IGROV1 cells arrested in the G2/M phase of the cell cycle could be observed for cells treated with 10 nM PTX (46.5% ± 3.4%, Figure 1b) compared to untreated control cells (25.2% ± 7.3%, Figure 1a). Furthermore, an increased sub-G1 population (18.2% ± 2.2%, Figure 1b) was detected during PTX treatment. Thereby, G0/G1 population decreased from 54.5% ± 2.5% (untreated cells) to 15.7% ± 1.2%. The treatment with 5 nM PTX resulted in 17.0% ± 9.3% cells being arrested in the G2/M phase of the cell cycle accompanied by an increased sub-G1 population (25.4% ± 4.8%, Figure 1c) compared to the control. The G0/G1 cell population decreased to 36.7% ± 13.6% compared to the untreated cells.

### *In vitro* cell growth inhibition upon ^177^Lu-DOTA-chCE7 and paclitaxel treatments

**Figure 2:**
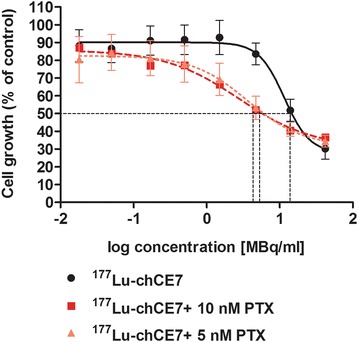
***In vitro*** **effects of single or combination treatments containing** ^**177**^ **Lu-DOTA-chCE7 and PTX on IGROV1 cell viability.** Determination of cell growth was assessed via MTT assays 120 h post treatment. Results are expressed as percentage of an untreated control.

In order to investigate whether PTX can sensitise IGROV1 cells towards subsequent ^177^Lu-DOTA-chCE7 treatment, cell growth inhibition upon single or combined treatments was examined by MTT proliferation assays using previously determined IC_50_ (10.0 ± 1.0 nM) and ½ IC_50_ (5 nM) of PTX as a basis for combination treatments. One-half IC_50_ was calculated based on the experimentally determined IC_50_ (Additional file 1: Figure S2). Treatment of IGROV1 cells with ^177^Lu-DOTA-chCE7 alone revealed an IC_50_ of 11.9 ± 1.9 MBq/ml (Figure 2). Combined treatment including PTX and ^177^Lu-DOTA-chCE7 resulted in decreased IC_50_ values of 3.5 ± 1.3 MBq/ml (^177^Lu-DOTA-chCE7 + 5 nM PTX) and 2.9 ± 1.2 MBq/ml (^177^Lu-DOTA-chCE7 + 10 nM PTX). In order to determine synergistic or additive effects, combination index (CI) calculations as published by Zhao et al. were used [36]. For a cell growth inhibition of 50%, the additional application of PTX (½ IC_50_) increases the cytotoxic effect of ^177^Lu-DOTA-chCE7 in a synergistic manner (CI < 1). In order to provide additional information about the long-term colony-forming ability of IGROV1 cells after mono- (^177^Lu-DOTA-chCE7) or combined (PTX + ^177^Lu-DOTA-chCE7) treatments, colony assays were assessed. Results indicate that the cytotoxicity of ^177^Lu-DOTA-chCE7 can be increased by the additional application of paclitaxel (½ IC_50_) 24 h prior to RIT (Additional file 1: Figure S3). Thereby, IC_50_ values decreased from 1.5 MBq/ml (^177^Lu-DOTA-chCE7) to 0.75 MBq/ml for combined treatment (PTX + ^177^Lu-DOTA-chCE7). Simultaneous administration or application of PTX 16 h after ^177^Lu-DOTA-chCE7 did not increase the cytotoxicity effects of RIT necessary for 50% cell growth inhibition.

### Combination of ^177^Lu-DOTA-chCE7 and PTX results in prolonged survival of human ovarian carcinoma-bearing nude mice

To investigate whether a combined treatment of ^177^Lu-DOTA-chCE7 and PTX results in delayed tumour growth and prolonged survival compared to monotreatments, a therapy experiment was performed in nude mice. Xenografts were generated by s.c. injection of IGROV1 human ovarian carcinoma cells into female CD1 nude mice.

**Figure 3:**
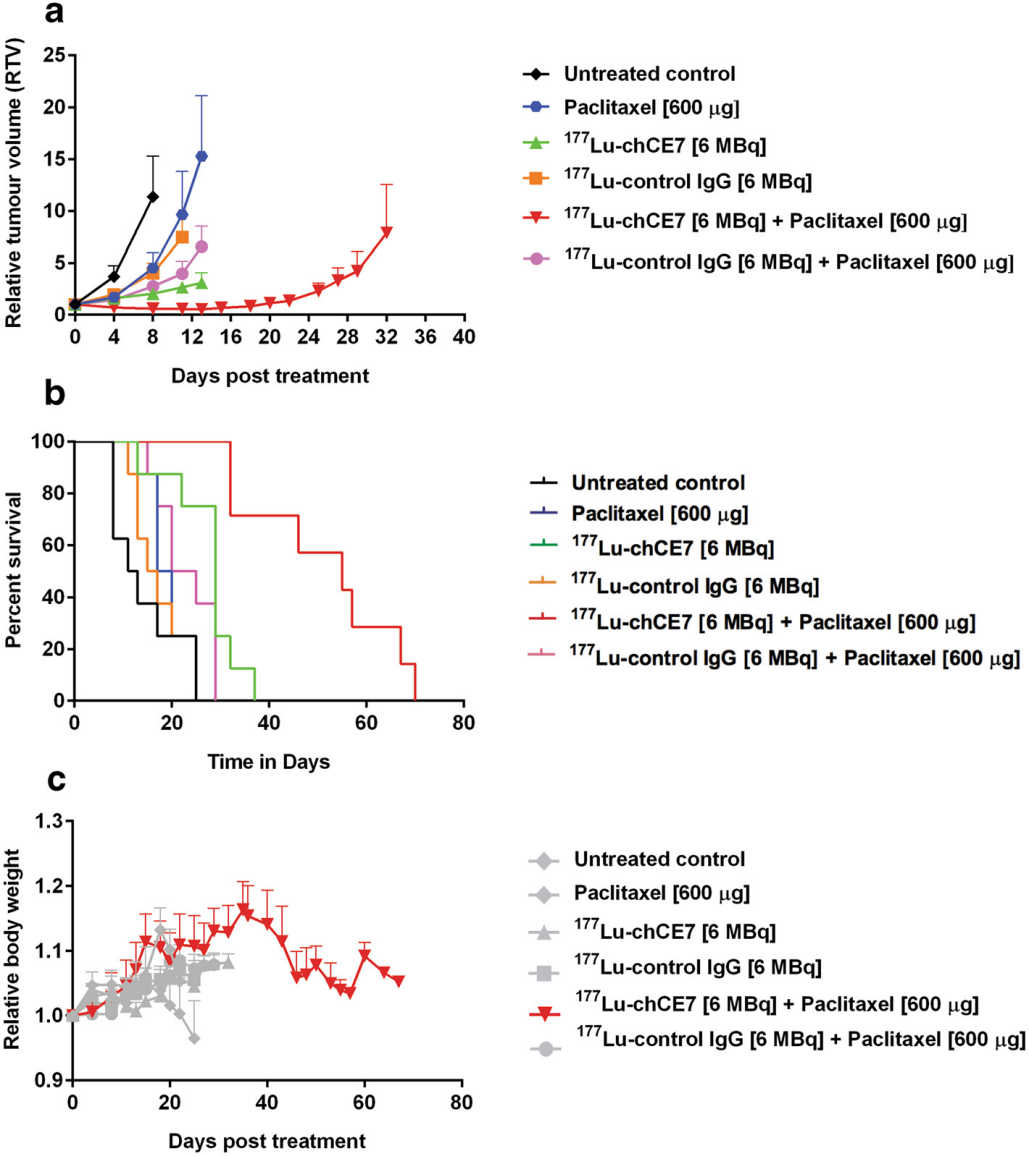
**Therapeutic efficacy of anti-L1CAM RIT in combination with PTX.** Tumour-bearing nude mice (*n* = 8) received ^177^Lu-DOTA-chCE7 (6 MBq, 50% MTA, i.v.) followed by PTX (600 μg, i.p.) 24 h later for combination therapies. Control mice received PBS, PTX or 6 MBq (25 μg) of ^177^Lu-labelled unspecific control IgG with or without PTX. **(a)** Mean relative tumour volume ± SD. Tumour growth curves were stopped when the first tumour in a treatment group reached 1,000 mm^3^. **(b)** Kaplan-Meier plots of the therapy experiment. **(c)** Development of body weight during therapy. Mean relative body weight ± SD.

Eight days post implantation of tumour cells, mice received an i.v. injection of 6 MBq ^177^Lu-DOTA-chCE7 (25 μg) followed by 600 μg PTX 24 h later. Control mice received either 6 MBq (25 μg) of a ^177^Lu-labelled unspecific control IgG or PBS. Mice were monitored two to three times a week with end point criteria set as weight loss >20% or increased tumour volume >1,000 mm^3^. Average RTVs, average RBWs and Kaplan-Meier survival plots were recorded. Tumour growth curves were stopped at the exclusion day of the first mouse in each group. Untreated control mice showed a fast increase in tumour volume from the beginning of the therapy experiment, indicating an uninhibited tumour growth (Figure 3a). The group that received the combination therapy (^177^Lu-DOTA-chCE7 + PTX) demonstrated the most pronounced delay in tumour growth. The mean RTV < 1 between days 4 and 18 post treatment start indicates tumour shrinkage. Complete tumour clearance or shrinkage below detection limits was observed in seven out of eight mice until day 13. During this time, a reduced tumour burden with a significant decrease in average RTV on day 13 was observable for mice treated with anti-L1CAM combination therapy compared to all other treatment groups (vs. ^177^Lu-DOTA-chCE7: *p* < 0.05; vs. PTX: *p* < 0.05; vs. ^177^Lu-DOTA-control IgG + PTX: *p* < 0.05). Additionally, no weight loss >20% or signs of distress could be observed at any time during the therapy experiment. Combination of ^177^Lu-DOTA-chCE7 and PTX resulted in a significantly prolonged overall survival compared to mice that received only ^177^Lu-RIT or PTX (vs. ^177^Lu-DOTA-chCE7: *p* = 0.0013; vs. PTX: *p* = 0.0002; Figure 3b). Thereby, median survival was increased from 18 days (PTX) and 29 days (^177^Lu-DOTA-chCE7) up to 55 days for the combination therapy. Additionally, a significantly prolonged survival could be shown for mice receiving anti-L1CAM combination therapy compared to untreated controls (PBS) and mice that received unspecific treatments (vs. PBS: *p* = 0.0001; vs. ^177^Lu-DOTA-control IgG: *p* = 0.0001; vs. ^177^Lu-DOTA-control IgG + PTX: *p* = 0.0002). No significant difference in overall survival could be observed between PTX monotreatment and the untreated control (*p* = 0.29).

The ^177^Lu-RIT alone increased overall survival significantly compared to mice that received only PBS (*p* = 0.0017). A regular increase in mean RBW for mice that received the combination of ^177^Lu-DOTA-chCE7 and PTX could be observed until day 37 post treatment start indicating no therapy-induced weight loss and/or signs of toxicity (Figure 3c). However, subsequent decrease in body weight correlates with reoccurring increased tumour burden.

### Comparative biodistributions and SPECT/CT imaging post ^177^Lu-DOTA-chCE7 and PTX administrations

**Figure 4:**
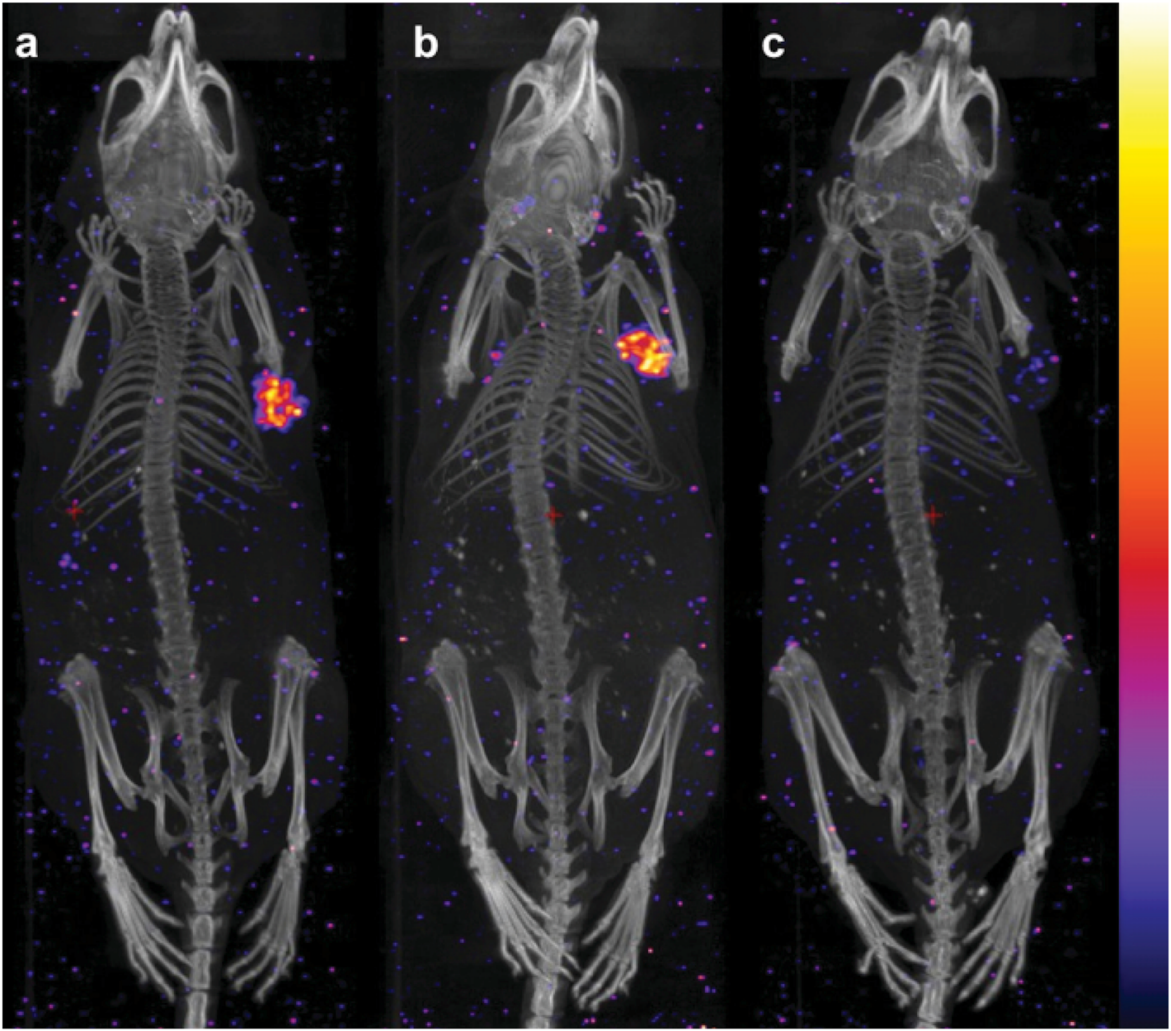
**Whole-body SPECT/CT images of IGROV1 xenografts.** CD1 nude mice were injected i.v. with 6 MBq of **(a)** ^177^Lu-DOTA-chCE7 or **(b)** ^177^Lu-DOTA-chCE7 and 600 μg PTX 24 h post RIC administration or **(c)** ^177^Lu-DOTA-control IgG. Images were taken 120 h post RIC injection (control 96 h).

Previously, Jang et al. [27] investigated the effect of PTX on the efficacy of ^90^Y-labelled B3 mAb in Le^y^ antigen-positive A-431 human epidermoid carcinoma xenografts. It was demonstrated that PTX significantly increased the accumulation and penetration of mAb B3 into the tumour microenvironment compared to the control, supporting synergistic effects of the combined therapy. In order to investigate whether PTX treatment influences the biodistribution of ^177^Lu-DOTA-chCE7, female CD1 nude mice (*n* = 4) were injected i.v. with 0.85 MBq ^177^Lu-DOTA-chCE7 14 days post s.c. implantation of IGROV1 tumour cells. Twenty-four hours post ^177^Lu-DOTA-chCE7 administration, one group of mice additionally received 600 μg PTX (i.p.). Control mice (*n* = 5) were injected with a non-binding ^177^Lu-DOTA-control IgG with or without PTX treatment. Accumulated radioactivity in tumour tissue was high after 72 h (49.6% ± 11.9%) for mice injected with ^177^Lu-DOTA-chCE7 compared to reasonably low uptakes for all non-targeted organs and the blood pool (<9%; Table 1). RIC uptake of 45.3% (±8.6%) per gram tumour tissue could be reached in mice that received successive ^177^Lu-DOTA-chCE7 and PTX administrations. Accumulated radioactivity in tumours did not differ significantly for both groups (*p* = 0.58), implying that PTX has no effect on the tumour uptake of ^177^Lu-DOTA-chCE7. Similarly, PTX had no influence on the accumulation of ^177^Lu-DOTA-chCE7 in all other organs and the blood pool. As expected, the tumour uptake of the ^177^Lu-DOTA-control IgG was low regardless of a following PTX administration. For ^177^Lu-DOTA-control IgG, we observed a longer retention time in the blood pool compared to ^177^Lu-DOTA-chCE7. Very low radioactivity in the bones indicates maintained stability of the ^177^Lu-DOTA complex after 72 h post injection. We further analysed the distribution of ^177^Lu-DOTA-chCE7 in subcutaneous IGROV1 tumour-bearing nude mice at 120 h post RIC administration via SPECT/CT. Injection of ^177^Lu-DOTA-chCE7 (Figure 4a) and combined administrations of ^177^Lu-DOTA-chCE7 and PTX (Figure 4b) show similar high uptakes of the RICs in the tumours located at the right shoulder. In both cases, almost no remaining activity in other non-targeted organs could be observed, matching the low accumulation percentages obtained from biodistribution studies of the earlier time point (72 h). Additionally, SPECT/CT images indicate that ^177^Lu-DOTA-chCE7 was equally distributed throughout the tumour regardless of a following PTX injection. Neither an uptake at the site of tumour implantation nor uptake in other organs could be detected 96 h post injection of ^177^Lu-DOTA-control IgG (Figure 4c).

**Table 1:** **Biodistribution of** ^**177**^ **Lu-DOTA-chCE7 and** ^**177**^ **Lu-DOTA-control IgG 72 h post RIC injection in nude mice bearing subcutaneous IGROV1 tumours (±PTX)**

**Organs**	^**177**^ **Lu-DOTA-chCE7**	^**177**^ **Lu-DOTA-chCE7 + PTX**	^**177**^ **Lu-DOTA-control IgG**	^**177**^ **Lu-DOTA-control IgG + PTX**
Blood	8.9 ± 2.8	6.9 ± 3.5	17.12 ± 3.6	13.3 ± 8.5
Heart	3.9 ± 0.8	3.3 ± 1.4	5.7 ± 2.9	3.9 ± 1.7
Spleen	7.0 ± 2.3	5.7 ± 1.0	5.0 ± 2.8	10.2 ± 6.1
Kidney	2.8 ± 0.3	2.4 ± 0.6	5.4 ± 1.9	4.6 ± 1.8
Stomach	0.4 ± 0.2	0.4 ± 0.1	0.8 ± 0.8	1.0 ± 0.4
Intestine	0.8 ± 0.2	0.7 ± 0.3	1.1 ± 0.3	0.4 ± 0.5
Liver	5.3 ± 1.8	5.7 ± 0.3	5.7 ± 2.5	6.7 ± 1.2
Muscle	0.9 ± 0.2	0.8 ± 0.2	1.1 ± 0.3	1.4 ± 0.6
Bone	1.5 ± 0.2	1.1 ± 0.5	1.9 ± 0.6	2.6 ± 0.5
Tumour	49.6 ± 11.9	45.3 ± 8.6	7.2 ± 2.3	9.1 ± 3.1

Results shown in %IA/g ± SD.

## Discussion

We have previously demonstrated that anti-L1CAM RIT using the mAb chCE7 is effective against small disseminated ovarian tumour nodules in a preclinical setting [25,37]. In this study, we asked for the first time if the efficacy of L1CAM-targeted ovarian cancer RIT can be further improved by the introduction of PTX into the therapy scheme. Therefore, the efficacies of ^177^Lu-DOTA-chCE7 and PTX monotreatments were compared to the combined treatment modality. We further evaluated if PTX influences the tumour uptake of ^177^Lu-DOTA-chCE7.

After adding PTX at IC_50_ to IGROV1 ovarian cancer cells, the number of cells being arrested in the radiosensitive G2/M phase of the cell cycle could be increased at 24 h after treatment start. In contrast, PTX at ½ IC_50_ showed no increase in G2/M phase arrested cells compared to an untreated control, suggesting that the applied concentration was not sufficient to induce cell cycle arrest. Nevertheless, for both concentrations, the appearance of sub-G1 populations was demonstrated.

These results agree with previous observations that low PTX concentrations (<10 nM) induced an increased amount of apoptosis without evidence for existent G2/M arrest compared to a control. This effect might be caused by the fact that lower PTX concentrations (<10 nM) do not completely saturate PTX binding sites in a part of the cell population, which in turn leads to progression of the cell cycle, chromosomal instability and induction of apoptosis [38]. For intermediate PTX concentrations (≥10 nM), saturation of microtubule binding sites may be further increased, leading to G2/M arrest and apoptosis [38]. However, in both cases, ^177^Lu-DOTA-chCE7 concentrations necessary to reduce cell viability to 50% of untreated controls could be reduced 3.2-fold by combination with low-dose PTX at ½ IC_50_ or 4.3-fold when combined with IC_50_ PTX intermediate dosages. For 50% cell growth inhibition, combination index calculations revealed that monotreatments (½ IC_50_ PTX + ^177^Lu-DOTA-chCE7) were combined in a synergistic manner.

Increased therapeutic efficacy of RIT upon combination with PTX has been previously shown by Jang et al. [27] when anti-Le^y^^90^Y-labelled mAb B3 and PTX combination therapy resulted in a significantly prolonged survival of human epidermoid carcinoma-bearing mice. Milenic et al. [34] demonstrated an increased therapeutic efficacy when α-particle-targeted radiation therapy (^213^Bi-trastuzumab) was combined with PTX in a human colon carcinoma tumour model. However, the radioisotope ^177^Lu is a more suitable candidate for RIT against smaller tumours that frequently appear in ovarian cancer. ^177^Lu shows an increased half-life of 6.7 days compared to ^213^Bi (45 min), thereby matching the slow pharmacokinetics of IgGs when injected intravenously. Furthermore, an intravenous application of the RIC might have advantages in terms of targeting distant metastasis beyond the peritoneal cavity. Thereby, ^177^Lu lower tissue penetration range (≈2 mm) is likely to be superior to ^90^Y in the treatment of small disseminated ovarian cancer tumour nodules [25]. Our animal studies demonstrated that combined application of PTX and anti-L1CAM ^177^Lu-DOTA-chCE7 led to an increased therapeutic efficacy in a xenograft model, resulting in a significantly prolonged overall survival. Thereby, PTX was administered 24 h post RIT in order to adapt peak concentrations of ^177^Lu-DOTA-chCE7 and PTX in the tumour. Results indicate that PTX has the ability to increase the cytotoxic effects on IGROV1 tumour cells induced by ^177^Lu-DOTA-chCE7. Mice that received only PTX monotreatment showed no significant difference in tumour growth delay and overall survival compared to untreated control mice, implying that only subcytotoxic PTX concentrations (600 μg, 31.6 mg/kg) were used. Observation of insignificant tumour suppression of PTX monotreatments compared to untreated controls is in line with previous studies demonstrating the limited subtherapeutic effect of low-dose PTX treatments [35].

Biodistribution studies demonstrated high tumour uptakes for the specific RIC correlating with low remaining levels of activity in the blood pool 72 h post RIC injection, regardless of an additional PTX administration. Thus, PTX did not influence the ^177^Lu-DOTA-chCE7 uptake in either way. As expected, tumour uptake of the control RIC with or without PTX application was very low, indicating only a non-specific accumulation. Low non-specific tumour uptakes were consequently accompanied by higher remaining levels of activity in the blood pool.

Jang et al. [27] demonstrated that higher PTX dosages (>600 μg) as well as larger mean tumour volumes at therapy start (≈200 mm^3^) showed decreased interstitial fluid pressure and increased blood vessel permeability resulting in higher RIC accumulation in the tumour. In our studies, increased tumour accumulation was not observed, supporting our assumption that cell cycle arrest in the G2/M phase played a major role during *in vivo* combination therapy. However, additional effects on the tumour microenvironment caused by PTX cannot be fully excluded, since cellular and tumour microenvironmental effects are known to complement each other [31].

Even though the therapeutic efficacy of ^177^Lu-DOTA-chCE7 was increased by the introduction of PTX into the treatment scheme, no weight loss or a decreasing number of white blood cells was induced (data not shown).

While the application of a third chemotherapeutic is thought to result in unjustifiable toxicities for patients, RIT has shown to be well tolerated with low toxicity levels. Therefore, RIT/paclitaxel/platinum-based chemotherapy combination offers an alternative treatment strategy that may improve the efficacy of a first-line platinum-paclitaxel treatment. Nevertheless, such an alternative treatment strategy has to be verified in large, randomised clinical trials. Since patterns of platinum resistance and mechanisms of action for microtubule-stabilising agents do not necessarily interact, patients with platinum-refractory, platinum-resistant disease or platinum-resistant relapse might benefit from a RIT/paclitaxel combination. Again, this has to be verified in clinical trials.

Malignant ascites are frequently occurring in patients with advanced ovarian cancer with only limited treatment options. So far, catumaxomab, a trifunctional mAb, is used for the effective treatment of EpCAM-positive tumour cells in the peritoneal cavity [39]. An effective treatment against L1CAM-positive tumour cells in the peritoneal cavity might therefore decrease the amount of free tumour cells (spheroids) potentially reducing the burden of occurring ascites.

## Conclusions

It is well known that patients suffer from severe side effects (e.g. neuropathy and myelosuppression) induced by high concentrations of PTX during treatment cycles [40]. Since RIT is more tolerable compared to high-dosage chemotherapies, a combination with radiosensitising agents like PTX might result in a similar or better therapeutic outcome, while lower effective therapeutic PTX dosages are necessary compared to mono-chemotherapeutic treatments. The introduction of PTX in the therapy scheme of L1CAM-targeted ^177^Lu-DOTA-chCE7 RIT may provide a potential clinical setup against residual ovarian tumour nodules after first-line tumour resection. Patients may therefore benefit from decreased side effects during therapy and possible increased therapeutic efficacy.

## Additional file

Additional file 1:
**Supplementary information.** The file contains discussion and figures on plasma stability test, determination of half-maximal inhibitory concentration (IC_50_) of paclitaxel, and *in vitro* cell growth inhibition upon ^177^Lu-DOTA-chCE7 and paclitaxel treatments via colony assay.
