# Supplementary material for: Paclitaxel improved anti-L1CAM lutetium-177 radioimmunotherapy in an ovarian cancer xenograft model
Source: EJNMMI Res. 2014 Oct 3;4:54. doi: 10.1186/s13550-014-0054-2 (PMC4452682; doi:10.1186/s13550-014-0054-2)
Supplement: Additional file 1: — Supplementary information. The file contains discussion and figures on plasma stability test, determination of half-maximal inhibitory concentration (IC50) of paclitaxel, and in vitro cell growth inhibition upon 177Lu-DOTA-chCE7 and paclitaxel treatments via colony assay. [file 13550_2014_54_MOESM1_ESM.docx]

**Additional file 1**

**Combination of anti-L1CAM lutetium-177 radioimmunotherapy and paclitaxel improved survival of nude mice bearing human ovarian cancer**

Dennis Lindenblatt^1^, Eliane Fischer^1^, Susan Cohrs^1^, Roger Schibli^1,2^ and Jürgen Grünberg^1^

^1^Center for Radiopharmaceutical Sciences ETH-PSI-USZ, Paul Scherrer Institut, Villigen PSI, Switzerland; ^2^Institute of Pharmaceutical Sciences, ETH Zürich, Switzerland

For correspondence or reprints contact: Jürgen Grünberg, Center for Radiopharmaceutical Sciences ETH-PSI-USZ, Paul Scherrer Institut, 5232 Villigen PSI, Switzerland;
Phone +41-(0)56-3102848.
Email: juergen.gruenberg@psi.ch

**Plasma stability Test**

In order to test the stability of ^177^Lu-labelled antibodies, RICs were incubated in human plasma at 37 ^0^C and analysed by FPLC size exclusion chromatography on a TSKgel G3000Wxl column (Tosoh Bioscience, Stuttgart, Germany). Flow rate of mobile phase (0.3 M NaCl, 0.05 M Na2HPO4, pH 6.2) was set to 1ml/ min.


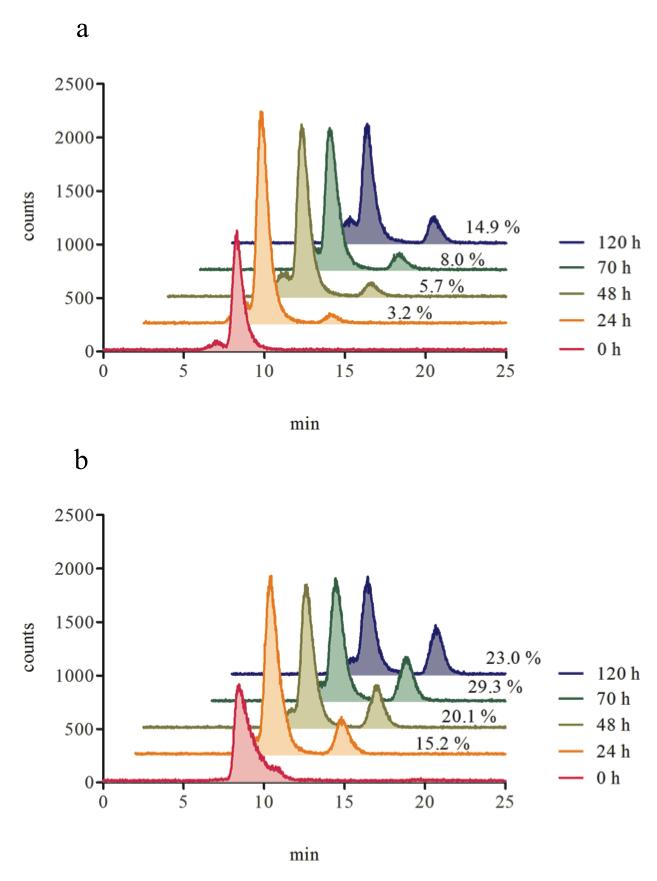


**Additional file 1: Figure S1:** Plasma stability after 0 h, 24 h, 48 h, 70 h and 120 h for a) ^177^Lu-DOTA-chCE7 and b) ^177^Lu-labelled control IgG.

**Determination of half-maximal inhibitory concentration (IC_50_) of paclitaxel**

In order to determine the PTX concentration nesseccary to reduce cell viability to 50% (IC_50_), IGROV1 cells were seeded in a 96 well plate and incubated for 24 h.

After adhesion cells were treated with PTX concentrations ranging from 0.5-500 nM for 24 h at 37°C. After washing with PBS cells were incubated 120 h at 37°C. Cell viability was determined when 20 μl of filtered 3-(4,5-dimethylthiazol-2-yl)-2,5-diphenyltetrazolium bromide (MTT) solution (5 mg/mL, Sigma-Aldrich) was added to each well followed by incubation for 2 h protected from light. Media was removed and the formed formazan crystals were dissolved in 200 μl dimethyl sulfoxide (DMSO). The absorbance (OD) was determined at a wavelength of 560 nm in a microplate reader (Victor X3, Perkin-Elmer). Results are expressed as percentage of viable cells compared to control. IC_50_ concentration was determined with 10 ± 0.95 nM.

###
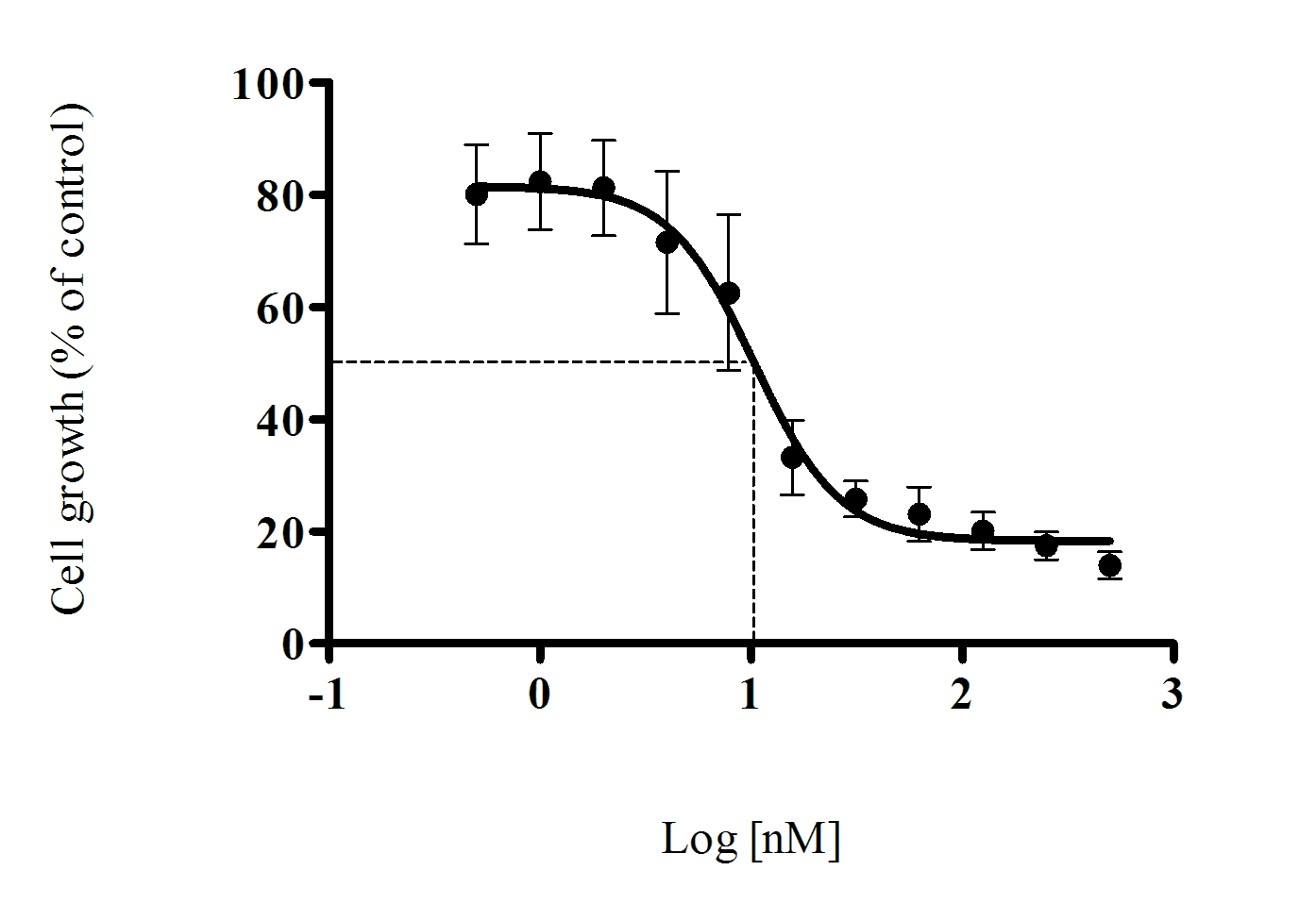
Additional file 2: Figure S2: Half-maximal inhibitory concentration (IC_50_) of paclitaxel against IGROV1 cells (10 ± 0.95 nM).

***In vitro* cell growth inhibition upon ^177^Lu-DOTA-chCE7 and paclitaxel treatments via colony-assay**

In order to determine the ^177^Lu-DOTA-chCE7 concentrations necessary to reduce cell viability to 50% (IC_50_) 250 IGROV1 cells were seeded in a 6 well plate and incubated for 24 h at 37 ^0^C. After adhesion, cells receiving combination treatment were incubated with the accordant ½ IC_50_ (0.1 nM) PTX concentrations for 24 h at 37 ^0^C whether 24 h before, simultaneously or 16 h post treatment with 1 ml (0.001-4.5 MBq/mL) ^177^Lu-DOTA-chCE7 (24 h at 37 ^0^C). Subsequently cells were washed and incubated in culture media at 37 ^0^C for 9 days. Colonies were stained with crystal violet (Sigma-Aldrich) and counted manually.

**

**

**Additional file 1: Figure S3:** *In vitro* effects of single or combination treatments containing ^177^Lu-DOTA-chCE7 and PTX on IGROV1 colony-forming ability. PTX was added 24 h prior, simultaneously or 16 h post RIT. Results are expressed as percentage of an untreated control.
